# MOF@PEDOT Composite Films for Impedimetric Pesticide Sensors

**DOI:** 10.1002/gch2.201900076

**Published:** 2020-01-08

**Authors:** Luciano D. Sappia, Jimena S. Tuninetti, Marcelo Ceolín, Wolfgang Knoll, Matías Rafti, Omar Azzaroni

**Affiliations:** ^1^ Instituto de Investigaciones Fisicoquímicas Teóricas y Aplicadas Departamento de Química Facultad de Ciencias Exactas Universidad Nacional de La Plata CONICET, CC 16 Suc. 4 La Plata B1904DPI Argentina; ^2^ CEST – Competence Center for Electrochemical Surface Technologies Konrad Lorenz Strasse 24 3430 Tulln Austria; ^3^ Austrian Institute of Technology Donau‐City‐Strasse 1 1220 Vienna Austria; ^4^ CEST‐UNLP Partner Lab for Bioelectronics Diagonal 64 y 113 La Plata 1900 Argentina

**Keywords:** conductive polymers, imazalil, impedance spectroscopy, metal–organic frameworks, PEDOT, pesticide sensors

## Abstract

Due to its deleterious effects on health, development of new methods for detection and removal of pesticide residues in primary and derived agricultural products is a research topic of great importance. Among them, imazalil (IMZ) is a widely used post‐harvest fungicide with good performances in general, and is particularly applied to prevent green mold in citrus fruits. In this work, a composite film for the impedimetric sensing of IMZ built from metal‐organic framework nanocrystallites homogeneously distributed on a conductive poly(3,4‐ethylene dioxythiophene) (PEDOT) layer is presented. The as‐synthetized thin films are produced via spin‐coating over poly(ethylene terephtalate (PET) substrate following a straightforward, cost‐effective, single‐step procedure. By means of impedance spectroscopy, electric transport properties of the films are studied, and high sensitivity towards IMZ concentration in the range of 15 ppb to 1 ppm is demonstrated (featuring 1.6 and 4.2 ppb limit of detection, when using signal modulus and phase, respectively). The sensing platform hereby presented could be used for the construction of portable, miniaturized, and ultrasensitive devices, suitable for pesticide detection in food, wastewater effluents, or the assessment of drinking‐water quality.

## Graphical abstract

Development of portable devices for on‐site evaluation of pesticide residues remains a topic of great interest worldwide. With this aim, a chemi‐impedimetric sensor for imazalil is presented, based on metal–organic frameworks (MOFs) nanoparticles incorporated into conductive poly(3,4‐ethylene dioxythiophene) (PEDOT) films. The impedimetric sensors are extremely sensitive to imazalil concentration in the range of 15 ppb to 1 ppm.

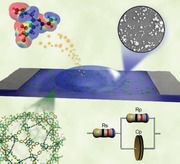

## 1 Introduction

**Scheme 1:**
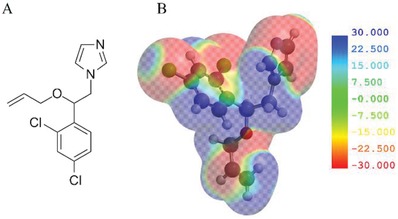
A) Imazalil molecular structure. B) Electrostatic potential map.

Fruits and vegetables are essential components of the human diet around the world providing important vitamins and nutrients required to maintain a good health status. In order to prevent the attack of insects and microorganisms after harvesting, the food industry uses pesticides to comply with quality standards during production and distribution.^1^ However, when above regulated levels, remnant pesticide in primary and derived agricultural products constitutes a health hazard.^2^ In the case of citrus fruits, a widely used fungicide for postharvest control of fungal pathogens is an azole fungicide known as imazalil (1‐(β‐allyloxy‐2,4‐dichlorophenethyl) imidazole), due to its specific action against green mold and Aspergillus (see **Scheme** **1**).^3^, ^4^, ^5^ Current European Union (EU) regulations (EC No. 396/2005) establish a maximum residual limit (MRL) for IMZ in fresh or frozen fruits ranging from 0.05 ppm (tree nuts, cereals, products of animal origin, among others) to 5 ppm in citrus fruits. A recent chemical study carried on liquid industrial effluents reported the presence of a wide range of residual pharmaceuticals and pesticides released to the environment. Regarding IMZ, a concentration of 0.032 and 0.040 ppm was detected,^6^ with potential harmful effects on both environment and persons.^7^

Gold standard methods for residual IMZ determination are high‐performance liquid chromatography (HPLC),^8^, ^9^ gas chromatography (GC),^10^, ^11^, ^12^ and liquid chromatography‐time‐of‐flight mass spectrometry (LC‐TOFMS).^13^, ^14^ The LOD (limit of detection) and LOQ (limit of quantification) reported for IMZ in liquid effluents using HPLC‐MS is 0.14 ppm.^6^ In the EU regulation, a detection limit of 0.05–0.1 ppm in food is reported using HPLC/GC‐MS.^15^ Recently, the use of UHPLC‐QTOF‐MS (ultra‐high‐pressure liquid chromatography‐quadrupole time‐of‐flight MS) as a powerful tool for IMZ and metabolites quantification in complex food samples was also reported.^16^

Although the performance of the above‐described methods is satisfactory, a major drawback is that its application is expensive and time consuming, and involves complicated preparation procedures, as well as qualified technicians to conduct the analyses. Therefore, alternative detection methods have been developed for IMZ determination in complex food samples, including enzyme‐linked immune adsorbent assays^17^ and capillary electrophoresis.^18^ The enzyme‐linked immunosorbent assay technique or ELISA technique results attractive because, while determination with the conventional HPLC method takes 4–5 h in citrus fruit samples, by using ELISA immunosorbent assay a single sample can be analyzed within 2 h and allows for high‐throughput screening.^19^

Different strategies towards the detection of analytes of interest such as IMZ, rely on the use of adsorbent porous solids (e.g., zeolites or carbons) assembled into devices which should be capable of transducing adsorbate load into a measurable signal. Having in mind that zeolites perform poorly in aqueous environments for the adsorption of uncharged or large organic compounds, while carbons excel with large nonpolar adsorbates; it is expected that IMZ would sit outside the range where good adsorption performance can be expected using either of the above‐mentioned adsorbents. This is why we envisioned the possibility of using an emergent class of porous materials known as metal–organic frameworks (MOFs) as novel adsorbent for the hereby proposed application.^20^, ^21^, ^22^ MOFs can be generally described as microporous crystalline solids with high surface area and very versatile surface chemistry, constructed from coordination of metal ions (or clusters containing metal ions), linked with multi‐dentate organic molecules.^23^, ^24^ The use of many MOFs was extensively explored regarding its possible applications in water‐based separations and chromatography,^25^, ^26^, ^27^, ^28^ provided that selected materials fulfill the crucial requisite of stability toward hydrolysis.^29^ The severity of this issue can be traced back to the labile nature of metal‐oxygen bonds present in many MOFs, which can ultimately lead to destruction of the coordination network, although particular process conditions (e.g., pH, temperature, or other solvents present) will ultimately dictate longevity.^30^ The advantageous features of different MOFs have been rapidly applied in the development of sensors.^31^, ^32^, ^33^, ^34^, ^35^, ^36^, ^37^ Based on the signal transduction possible for many configurations, sensors reported can be described as optical,^38^, ^39^ electrochemical,^40^, ^41^, ^42^ mechanical,^43^ photoelectrochemical,^44^, ^45^, ^46^, ^47^ or combined‐miscellaneous.^48^ Among MOFs, MIL‐101(Cr) is a well‐known material robust toward exposure to aqueous environments. This porous material can be described as built from the coordination of Cr(III) ions and H_2_BDC (benzene‐dicarboxylic acid) linker, which belongs to a subclass named after the acronym of Material from the Institute Lavoisier (MIL).^49^ MIL‐101(Cr) features very high surface areas (up to 5900 m^2^ g^−1^) depending on the synthesis conditions and activation procedures used. In addition, MIL‐101(Cr) features high thermal and chemical stability, and can be assembled into functional and stable thin films, as it was shown recently.^50^ Moreover, desired chemical moieties can be placed on MIL‐101(Cr) pore surface in a quite straightforward way by using conveniently modified BDC‐X linkers (with ‐X, e.g., ‐NO_2_, ‐NH_2_, or ‐SO_3_H). Such added moieties, although will naturally take up some of the available pore space, confer interesting possibilities for tailored adsorbate affinity, surface polar character modulation, or chemical functionalization. Specifically, it was already reported that the introduction of ‐NH_2_ moieties to yield MIL‐101‐NH_2_(Cr) material, which provides stronger interactions with water molecules, constitutes an improvement over MIL‐101(Cr) when aiming for water‐based liquid adsorption applications.^51^ Due to the above discussed interesting features and to recent developments which allow avoiding the use of highly corrosive and toxic hydrofluoric acid in the synthesis procedure,^52^ MIL‐101‐NH_2_(Cr) was selected for IMZ adsorbent in the hereby proposed electrochemical sensing application.^53^, ^54^

Electrochemical sensors solely based on MOFs are rare due to their proverbial poor electrical conductivity (although recently reported conductive MOFs may soon expand the scope of possible applications).^55^ Straightforward methods available for the construction of composites with electroactive materials (e.g., conductive polymers or CPs) have paved the way for developing novel sensors,^56^, ^57^ where the advantageous features of both components can be combined in a synergistic fashion.^58^, ^59^, ^60^, ^61^ Among the CPs already reported for its use in MOF composites, poly(3,4‐ethylene dioxythiophene) or PEDOT has great potential for its use in technological applications due to both, high biocompatibility, conductivity, and versatility regarding its integration in composite films. However, there has been few reports on such use, e.g., MOFs were used as easy‐to‐remove porous templates for PEDOT yielding ultimately a mesostructured conductive material,^61^ or also used to yield composites with graphene oxide to create flexible supercapacitors.^62^, ^63^ To the best of our knowledge, there were only two reports dealing with the use of MOF‐PEDOT composites for sensors. One of the reported approaches uses a porphyrin‐MOF as electrocatalytic coating over PEDOT nanotubes in order to achieve porous electrodes with high selectivity towards dopamine with linear response in the micromolar range, and sub‐micromolar detection limit.^64^ The other mentioned article reports on the use of MIL‐101(Cr) MOF as a porous host where different loadings of EDOT were polymerized with a vapor‐phase oxidant for application towards chemiresistive NO_2_ sensing as model system.^65^

In the present work, we introduce a novel strategy towards IMZ impedimetric sensing in aqueous environments by using label‐free MIL‐101‐NH_2_(Cr)@PEDOT composites. The design is based on a spin‐coating procedure without the need of using primer over a low‐cost nonconductive poly‐ethylene terephthalate (PET) substrate. Thorough characterization was conducted, and the capability for IMZ sensing using impedance spectroscopy was explored. It was determined a remarkable operational range of 0.015–1 ppm IMZ concentration, and an LOD of 1.6 and 4.2 ppb depending on the use of impedance modulus or phase, respectively.

## 2 Results and Discussion

Oxidative chemical deposition is a versatile technique widely used for the construction of functional surfaces. It involves the mixing of an oxidant agent (iron(III) tosylate), butanol, and pyridine as polymerization retardant. Then, desired monomer is added, and the solution is ready to be spin‐coated over selected substrate. In previous works, the described technique was used to produce mechanically robust, stable films with homogenous thickness, over glass, gold, silicon, plexiglass, and PET substrates. The possibility of adding extra polymeric components to the film adds further complexity degree, e.g., poly(allylamine), provides amine anchoring sites available for the attachment of biomolecules (e.g., enzymes, lectins, among others).^66^

On the other side, electric transport properties of thin films (2D nanomaterials) can be strongly affected by the presence of ions and different solvents on the solid–liquid interface. In the case of ZnO thin films, e.g., impedance spectroscopy was used to elucidate the conduction path mechanisms according to the charge of the ions presents in the solutions positioned on the surface. The addition of deionized water (DIW), phosphate‐buffered saline (PBS) buffer, glucose oxidase in PBS or glucose was observed to affect conduction mechanisms, changing the impedimetric response up to several orders of magnitude. Moreover, the real and imaginary parts of the electrical impedance can be used to model and determine the effect of ions present in the conduction mechanisms operating.^67^

### 2.1 Characterization

**Figure 1:**
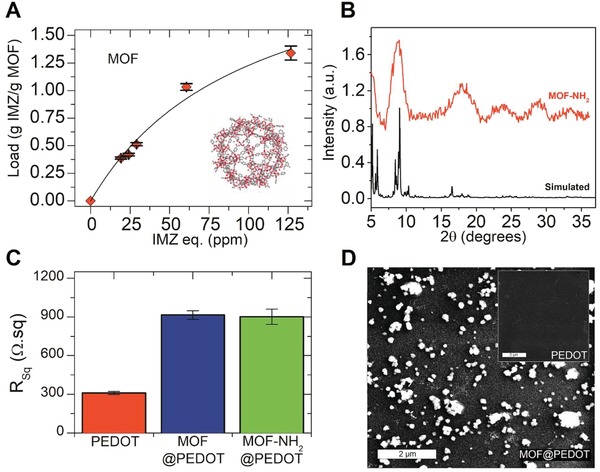
A) Adsorption equilibrium isotherm for IMZ on native MOF. B) Diffraction patterns for native and amino‐substituted MOF in comparison with the theoretical diffraction pattern for the native MOF. C) Sheet resistance of the PEDOT and MOF‐NH_2_@PEDOT thin films on PET substrates. D) SEM micrographs of the MOF‐NH_2_@PEDOT composite films and the bare PEDOT thin film (inset), both deposited on PET foils (scale bar is 2 µm).

**Figure** **1**A displays adsorption isotherm for IMZ on MIL‐101(Cr) MOF, which was used as porous matrix and demonstrate the adsorbate–adsorbant affinity (see also *Z*‐potential experiments discussed below), e.g., it features remarkably high final maximum loading of 1.3 g g^−1^ MOF. (See Figure S1 in the Supporting Information for experiments on the characterization of adsorption kinetics.) Moreover, taking into account both measured MIL‐101(Cr) Brunauer–Emmett–Teller surface area available, pore volume values (2700 m^2^ g^−1^ and 1.8 cm^3^ g^−1^), and the maximum IMZ loading calculated from Langmuir isotherm fit (see Figure S2 in the Supporting Information), the packing density can be estimated to be ≈0.75 g cm^−3^. Although lower than 1.35 g cm^−3^ IMZ bulk density, is remarkably high and points toward considerable pore accessibility even when using aqueous environments and non –NH_2_‐substituted material, making it a suitable adsorbent for the intended purposes. Additional evidence of the specific interaction between IMZ and the porous component on the MOF@PEDOT films was obtained by performing *Z*‐potential measurements. A variation from −22 to −13 mV in *Z*‐potential was detected when IMZ concentration was increased from 0 to 1 ppm (see Figure S3 in the Supporting Information). This result reveals that electrostatic forces play a key role on IMZ binding, causing an effect on the impedance response (see below experiments for IMZ detection).

In Figure 1B, diffraction patterns of MOFs synthesized are presented and an acceptable agreement with the calculated pattern for the cubic structure of MIL‐101(Cr) can be observed.^54^, ^68^ Finally, thermogravimetric analysis (TGA) experiments presented in Figure S4 in the Supporting Information showed the characteristic thermal stability of the polymeric matrix up to 300 °C.^69^ Weight loss observed between 150 and 300 °C for MIL‐101(Cr) is ascribable to residual organic linker entrapped in the inner MOF porosity.^70^ The experiments carried for characterization allow to confirm the successful synthesis of MOFs.

The strategy followed for the integration of PEDOT and MOF was to use an oxidant solution containing iron (III) tosylate ions and pyridine dissolved in butanol before the film deposition. Then, EDOT monomer was added to the solution with a final MOF suspension prepared for a concentration of 2 mg mL^−1^, and then films were immediately deposited by spin coating (polymerization was achieved by heating spin‐coated films by direct contact with a surface at 50 °C). Integration of MOF crystals into the conductive matrix was first assessed by measuring the sheet resistance of deposited nanofilms over PET with a four‐point probe (4PP) conductivity measurements. As can be observed in Figure 1C, MOF addition (hereby referred to as MOF@PEDOT and MOF‐NH_2_@PEDOT) increased the sheet resistance approximately three times when compared with the bare PEDOT layer on PET. The final concentration of MOFs in the oxidant solution was optimized in order to obtain the highest resistance while maintaining homogenously distributed crystals in the polymer film, thus preventing possible short‐circuits through the CP after the interaction of the analyte IMZ with the MOFs structures.

A PEDOT layer was deposited on a silicon substrate in order to perform thickness determination via XRR (X‐ray reflectivity) experiments. The thickness of a smooth, thin transparent film can be analyzed by measuring X‐rays reflection and critical angle.^71^ This is why XRR technique can be used to quantify film thickness for bare PEDOT but it is not suitable for typically nonsmooth (thus having strong scattering effects) MOF@PEDOT composite films. Figure S4B in the Supporting Information shows the XRR experiment from which a 20 nm thickness value can be calculated analyzing angular distance between reflection minima. Resistance of bare PEDOT and MOF@PEDOT composite films is presented in Figure 1C. In the case of bare PEDOT, the sheet resistance was 300 Ω sq^−1^, while the addition of both native and amino‐substituted MOFs increased the sheet resistance approximately three times (900 Ω sq^−1^), as expected upon the addition of a nonconductive material. This result was in agreement with similar PEDOT films deposited by spin coating on cyclic olefin copolymer substrates and the same formulation with sheet resistance of 351 Ω sq^−1^.^72^ Scanning electron microscope (SEM) images of MOF‐NH_2_@PEDOT and PEDOT film deposited on PET foils are presented in Figure 1D.

In order to gain further insight into the consequences for film integrity of the already demonstrated MOF‐IMZ affinity, MOF‐NH_2_@PEDOT composite films were exposed to high concentrations of IMZ (200 ppm IMZ in 0.1 m KCl for 1 h). After contact time elapsed, SEM micrographs revealed that some of the MOF crystallites were detached from the film (probably due to the above discussed electrostatic interactions), thus evidencing the presence of MOFs crystals inside the polymer film (Figure S7 in the Supporting Information). It is important to highlight that such effect was not present when the electrodes were incubated with IMZ in the operational range hereby explored for the impedimetric sensor (0.015–1 ppm).

### 2.2 Impedance Spectroscopy as a Tool to Elucidate Conduction Mechanisms Operating on PEDOT and MOF@PEDOT Composite Films

Impedance spectroscopy experiments were performed in order to determine conduction mechanisms operating in PEDOT and MOF@PEDOT composite films upon addition of aqueous solutions on their surface. Afterward, MOF@PEDOT composite electrodes were exposed to IMZ solutions in order to demonstrate that the specific interaction between IMZ and MOF units introduces perturbations in the conduction properties of the porous films.

**Figure 2:**
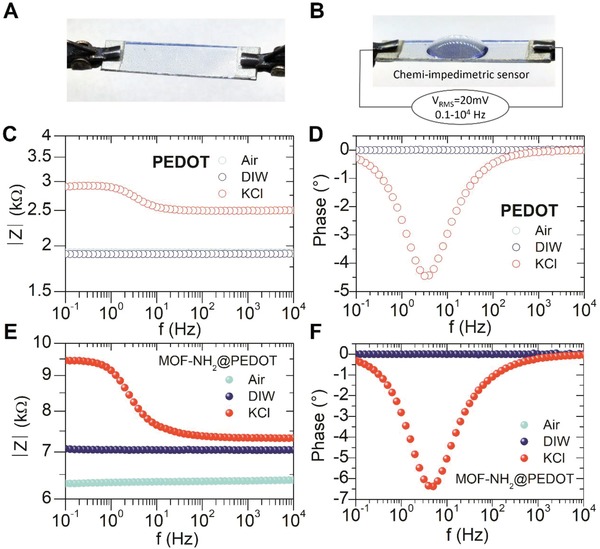
A,B) Experimental setup for the impedance experiments applying a 20 mV AC sinusoidal signal from 0.1 Hz to 10 kHz of a MOF‐NH_2_@PEDOT composite deposited on a PET substrate. The module and phase of the electrical impedance in air, DIW, and 0.1 m KCl are shown for C,D) the PEDOT and E,F) MOF‐NH_2_@PEDOT composite films.

**Scheme 2:**
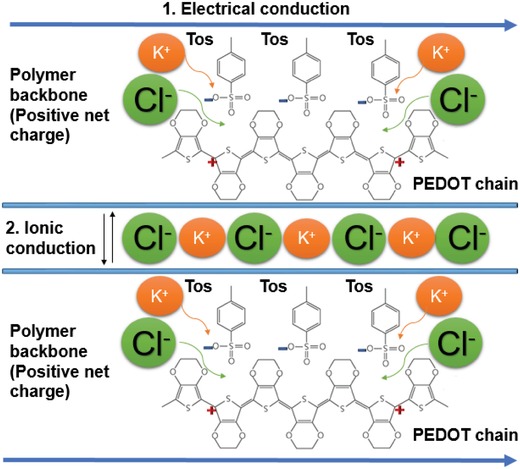
Conduction mechanisms inside the PEDOT film upon the addition of 0.1 m KCl.

The experimental setup used can be observed in **Figure** **2**A. A 20 mV_RMS_ AC voltage was applied on bare PEDOT films in order to determine the contribution of silver contacts and wiring. The results obtained are shown in Figure 2C,D, in terms of the modulus and phase of the impedance measured with a frequency sweep from 0.1 Hz to 10 kHz. Impedance response obtained in air (empty light blue circles) showed a pure resistive contribution, indicating that electrical contacts, wires, and metal/polymer junctions are not related to changes in the imaginary part of the impedance observed upon the addition of ionic solutions for the relevant frequency range explored (see below). For DIW, 40 µL droplet was added to the PEDOT surface, and the electrical impedance remained pure resistive (empty blue circles in Figure 2C,D); thus allowing to rule out an effect of PEDOT hydration (H^+^/OH^−^ ions) on the film's transport properties. On the contrary, when adding KCl solution, K^+^ and Cl^−^ ions were observed to interact with PEDOT films, as reflected by detected changes in the resistive component of the impedance, and the introduction of a resistive–capacitive behavior. There are both electronic (through the polymer backbone), and ionic (between the polymer chains) conduction mechanisms in PEDOT films, which are reflected in the impedance response obtained (see **Scheme** **2**).

The electronic conduction through the polymer backbone can be affected by the interaction with ions in contact solution. Chloride is frequently used as a dopant (or counter‐anion) of PEDOT synthesized via chemical oxidative and vapor‐phase polymerization,^73^ and the results hereby obtained suggest that it is possible that an exchange between Cl^−^ ions from the solution and tosylate anions from PEDOT can occur to some extent. It is also known that small dopant molecules can leach from the CP film and being gradually replaced by small ions from the electrolyte support solutions and additionally, the CP backbone can undergo redox reactions which will affect conductivity.^74^ All the above‐mentioned processes and effects can be used to rationalize the detected increase on the resistive component of the electrical impedance in the PEDOT film upon exposure to KCl, as show in Figure 2C,D.

The other mentioned conduction mechanism present (ionic) is associated with charge accumulation induced by ions near the depletion layer between polymer chains, and results in a large diffusion capacitance. It is known that the transport mechanism of ions in a polymer film can be either pure diffusional or incremented by electromigration (highly dependent on the magnitude of applied electric fields).^75^ For low voltages, only ion diffusion needs to be considered, while if voltages between 1 and 10 V are used then electromigration phenomena occur (with possible applications in the design of cation pumps, e.g., PEDOT:PSS (polystyrene sulfonate) films).^76^, ^77^

As is shown below, it is possible to model impedance response upon the addition of different solutions in order to elucidate the different conduction mechanisms operating for PEDOT and MOF@PEDOT composite films. For the impedance measurements presented in Figure 2, the response was modeled as composed by a pure resistive component associated with the electronic conduction, and a resistive/capacitive response attributed to charge accumulation in the polymer chains interfaces. The impedance response obtained for MOF‐NH_2_@PEDOT composite films is shown in Figure 2D,E. For this system, the observed increase on the resistive component can be attributed to the presence of MOF which agrees with 4PP resistance measurements carried on air. After exposure to DIW, resistive component of the impedance module of the films was observed to increase. This change can be attributed to film hydration triggered by increased hydrophilicity conferred by MOF units present, while phase response was observed to remain unchanged.^51^, ^78^ Finally, when a KCl droplet was added, the capacitive response observed for MOF‐NH_2_@PEDOT composite was higher than corresponding to bare PEDOT films (with similar effect for MOF@PEDOT composite, see Figure S5 in the Supporting Information). The above discussed enhancement of impedance response after incorporation of MOF‐NH_2_ can be understood in terms of the expected increased hydrophilic character of the composite.^79^ The porous and hydrophilic features of the MOF‐NH_2_ could lead to an increased contact area between the conductive PEDOT and the ionic solutions.

### 2.3 Impedance Response of the Films Upon the Addition of KCl and the IMZ Pesticide

**Figure 3:**
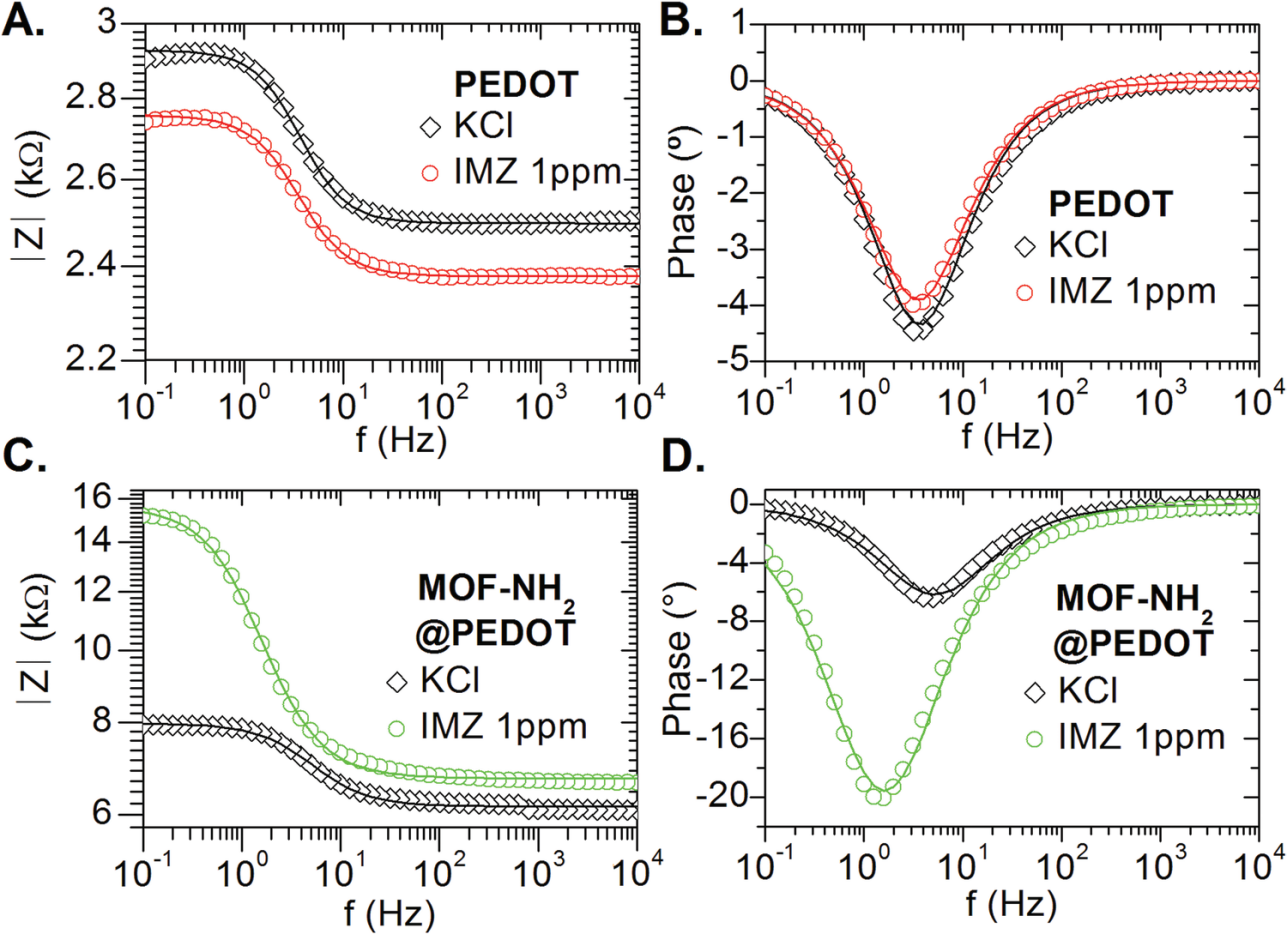
Experimental data (circles) and corresponding fitting (continuous lines) of the impedance module and phase response for the impedimetric sensors upon the addition of KCl 0.1 m and 1 ppm IMZ. Samples: A,B) PEDOT, C,D) MOF‐NH_2_@PEDOT. The equivalent electrical circuit used for modeling the impedance module and phase is described in the Supporting Information.

The electrical impedance of MOF‐NH_2_@PEDOT and bare PEDOT films was measured after the addition of 40 µL droplet of 1 ppm IMZ and KCl solutions. The transport properties through the conductive films were evaluated by applying a 20 mV_RMS_ AC voltage signal after the addition of 0.1 m KCl and 1 ppm IMZ in 0.1 m KCl. The impedance responses obtained are presented in **Figure** **3** for both, modulus and phase. For bare PEDOT films (Figure 3A,B), the addition of 1 ppm IMZ causes a 5.5% decrease on impedance modulus (|*Z*|0.2 Hz) and 10.8% decrease on phase (ϕ), as compared to the response obtained for 0.1 m KCl. The MOF‐NH_2_@PEDOT composite film (Figure 3C,D) reflected an opposite behavior, exposure to 1 ppm IMZ promoted an increase on the impedance modulus at 0.2 Hz of 89.7%, while the minimum of phase value showed a 213% increase. As a reference, the impedance response was also evaluated for the MOF@PEDOT composite, obtaining lower change percentage both in modulus (+38%) as well as in phase (+70%) upon the addition of 1 ppm IMZ (see Figure S5 in the Supporting Information).

The electrical impedance responses obtained were modeled in order to elucidate interaction mechanism between solutions and both PEDOT, and MOF@PEDOT composite films using the software Zview 3.0 (©Scribner Associates, Inc.). The equivalent circuits were determined to consist in a parallel RC element (Rp and Cp) connected in series with a resistance Rs (see Scheme S1 in the Supporting Information). Rs represents the resistive contribution of wiring, silver contacts, and the electronic conduction through PEDOT polymer. The RC parallel circuit can be associated with the interaction between ions in the droplet with polymer backbone. Capacitance response was modeled using a constant phase element (CPE), which is a nonideal capacitance that reflects inhomogeneities and defect areas on the surface and considers frequency dispersion of the capacitance value. Values higher than 0.8 of the CPE‐P factor (*f*) indicate a closely capacitive behavior, while a value of *f* = 1 corresponds to an ideal capacitor.^67^, ^80^ Additional details of the electrical model and experimental data fitting are presented in Section S3 in the Supporting Information, Figure S6 in the Supporting Information depicts the changes of the electrical equivalent elements upon the addition of the control and pesticide solutions.

Percentual changes in the passive elements from the equivalent electrical circuit used for modeling the impedance module of PEDOT, MOF@PEDOT, and MOF‐NH_2_@PEDOT films after the addition of 1 ppm IMZ are presented in Table S3 in the Supporting Information. For the MOF‐NH_2_@PEDOT film, addition of 1 ppm IMZ dramatically increases Rp value in 394%, while capacitance Cp decreased 22%. The parameter Rs remains almost unchanged (slight 7% increase), when compared to the initial resistance measured in 0.1 m KCl solution. For MOF@PEDOT films, addition of 1 ppm IMZ causes an increase of 21% in Rs, and 139% in Rp, while Cp decreased 27%.

**Figure 4:**
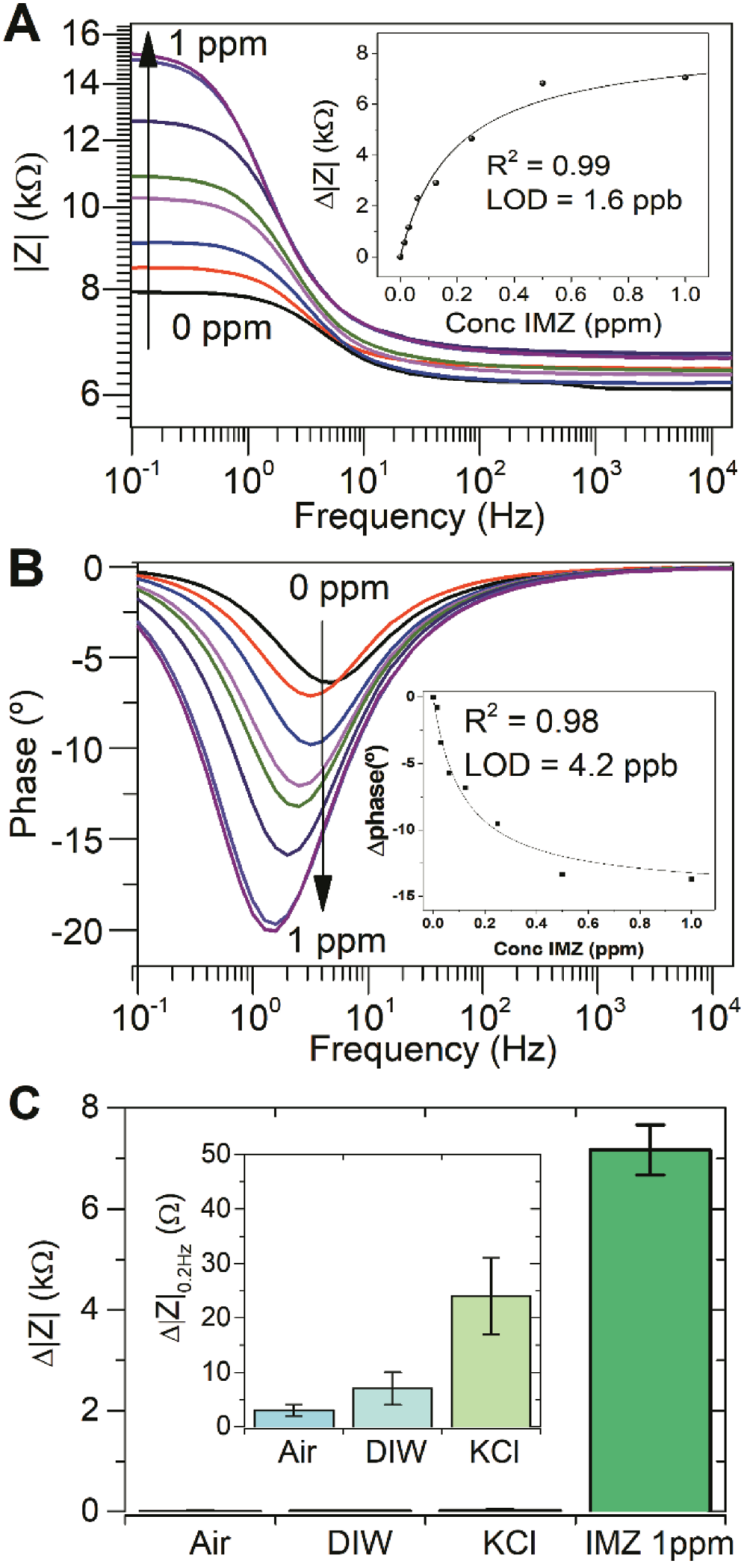
Experimental data obtained from the A) impedance module and B) phase of an MOF‐NH_2_@PEDOT films after the addition of IMZ at different concentrations (0.015, 0.03, 0.06, 0.12, 0.25, 0.5, and 1 ppm). In the insets, the calibration plots are obtained from the difference of each parameter with its blank obtained for three different samples. In (C), the relative change of the impedance modulus for the MOF‐NH_2_@PEDOT film is compared (air, DIW, 0.1 m KCl and 1 ppm IMZ in 0.1 m KCl). Error bars represent the standard deviation of three independent measurements.

Impedance modulus and phase responses were obtained for the MOF‐NH_2_@MOF composite film after sequential addition of increasingly higher IMZ concentrations (0.015–1 ppm). **Figure** **4**A,B shows the results obtained and insets depict calibration plots, featuring excellent hyperbolic correlation coefficient for both, phase (*R* ^2^ = 0.98) and impedance modulus (*R* ^2^ = 0.99). Furthermore, the lower LOD values obtained for phase and module of the impedance for IMZ were 4.2 and 1.6 ppb, respectively; approximately 1000 to 3000 times lower than the citrus MRLs established by the EU regulations (5 ppm). In the case of other vegetables or fruits, the MRLs are established by LOD of the analytical technique used (e.g., HPLC) as 0.05–0.1 ppm (see Tables S1 and S2 in the Supporting Information).^15^ It is important to highlight that the high affinity of MOF‐NH_2_ for IMZ allowed us to obtain an impedance response in modulus ≈300 times higher for IMZ 1 ppm when compared with the response using 0.1 m KCl (see Figure 4C). Furthermore, we carried out studies to evaluate interference upon the addition of two analogous fungicides. Fluconazole (FLU) and thiabendazole (TBZ) (two common post‐harvest pesticides) were added up to a final concentration of 1 ppm, and experiments were carried using the same experimental conditions than for IMZ. The analytical response obtained for control experiments and IMZ can be observed in Figure S9 in the Supporting Information. The response for the variation of modulus of impedance at 0.2 Hz frequency represents only a small fraction of the corresponding signal obtained for IMZ (0.5% (TBZ) and 8% (FLU)).

In Figure S8 in the Supporting Information, MOF@PEDOT composite films response was evaluated after exposure to IMZ solutions in the same concentration ranges as used for MOF‐NH_2_@PEDOT films. The calibration plot for IMZ measured on the MOF@PEDOT composite film showed poor correlation compared to MOF‐NH_2_@PEDOT presented above (*R* ^2^ = 0.795 for module and *R* ^2^ = 0.654 for the phase).

The impedimetric sensor showed LOD comparable with reference analytical methods widely reported (see **Table** **1**), but with the added great potential of portability and implementation on disposable plastic electrodes.

**Table 1:** Compilation of analytical methods for imazalil detection

Method	Limit of detection (LOD) in ppm
HPLC‐MS	0.014^6^
HPLC/GC‐MS	0.01–0.05^15^
HPLC‐UV detector	0.01^5^
ELISA	0.110–3^19^
Capillary electrophoresis	0.1–1^81^
White light reflectance spectroscopy	0.06^82^
Chemi‐impedimetric sensor	0.0016–0.0042 (this work)

## 3 Conclusions

In summary, we have synthetized MOFs@PEDOT composite films through a chemical‐oxidative polymerization and spin coating over low‐cost PET substrates without the use of primers. Conductive properties of films were studied by impedance spectroscopy upon the addition of different solutions. The specific interaction/specificity for the pesticide IMZ was exploited for the construction of chemi‐impedimetric sensors. The impedance response was modeled in the 0.1–10 kHz frequency range, and differences in the observed conduction mechanisms upon addition of solutions used were discussed. In the case of MOF‐NH_2_@PEDOT composite, the operational range of the sensor was evaluated for IMZ in the 0.015–1 ppm concentration range, with LOD of 0.0016 and 0.0042 ppm for the impedance modulus and phase responses, respectively. The hereby proposed method is suitable for assays carried under the required limits established by EU (0.01–5 ppm IMZ concentration). Furthermore, the high adsorption capacity and affinity of MOFs combined with conductive properties of PEDOT films can be tuned for the development of low‐cost sensors with high sensitivity, specificity with a great potential for miniaturization.^12^

## 4 Experimental Section

#### Synthesis of MIL‐101(Cr) and MIL‐101(Cr)‐NH_*2*_

Native and amino‐substituted MIL‐101 MOFs were prepared according to the already published procedures.^68^ Briefly, H_2_BDC (0.166 g, 1 mmol) or NH_2_BDC (0.181 g, 1 mmol) was added to TMAOH (tetramethylammonium hydroxide) 5 mL, 0.05 mol L^−1^, and ultrasonicated at room temperature for 10 min. Subsequently, Cr(NO_3_)_3_⋅9H_2_O (0.4 g, 1 mmol) was added to the mixture (pH of the resulting solution was measured to be 6.0–6.5) and further ultrasonicated for 20 min. The resulting solution was transferred into a Teflon‐lined autoclave and heated at 190 °C for 24 h. After slowly cooling to room temperature, the product was washed and centrifuged using DIW (2 × 30 mL), and EtOH (2 × 30 mL). The samples were then dried under vacuum at room temperature for activation before use (160 °C, 12 h).

#### Deposition of MIL‐101(Cr)@PEDOT Composite Thin Films

PEDOT films were deposited by in situ polymerization of the monomer solutions spin‐coated on PET sheets (3 cm × 3 cm × 0.05 cm), following the previously reported protocol with minimal modifications.^66^ Before polymerization, the plastic substrates were cleaned with pure ethanol and DIW, and dried under N_2_ flow. The oxidant solution consisted a mixture of iron (III) *p*‐toluenesulfonate 40% wt in butanol (C‐B 40 V2, Clevios, Heraeus Deutschland GmbH), butanol (ACS, Merck), and pyridine (ACS, Biopack). The PEDOT control samples were prepared by diluting the oxidant solution 1:1 with butanol and adding 7 µL of the monomer EDOT (97%, Sigma‐Aldrich) per 1 mL of the mixture. The as‐prepared reaction solution was mixed in a vortex, filtered (0.2 µm, Merck), and deposited on PET substrates by spin coating (WS‐650MZ‐23NPP, Laurell) using the resultant mixture at 750 rpm for 1 min with an acceleration of 500 rpm min^−1^. Finally, substrates were heated at 50 °C for 15 min to accelerate polymerization, immersed in DIW for 5 min, and dried under N_2_ flow.^66^, ^83^, ^84^

For the native and amino‐substituted MOF@PEDOT composite synthesis on PET substrates, 20 mg of MIL‐101(Cr) or MIL‐101(Cr)‐NH_2_ crystallites were first suspended in 5 mL butanol. The stock solutions were homogenized on a vortex and ultrasonicated (2200ETH S3, Sonica) for 10 min. Then, an aliquot of the MOF suspension was added to the oxidant solution for a final concentration of 2 mg mL^−1^. Finally, the EDOT monomer was added and the films were formed by spin coating as previous0ly described.

#### Characterization of the Composite Films Sheet Resistance

Sheet resistance measurements were measured using a TEQ‐03 potentiostat with a four‐point probe (4PP) accessory provided by the manufacturer (NanoTeq, Argentina). For each sample, five locations were measured and the average value was reported.

#### WAXS/XRR

Crystalline structure of MOFs was confirmed and PEDOT film thickness was assessed using wide‐angle X‐ray scattering (WAXS) and XRR configurations, respectively. Experiments were performed at INIFTA, on an XEUSS 1.0 HR (XENOCS, Grenoble) apparatus equipped with a Pilatus 100 K detector (Dectris AG, Switzerland) (pixel size of 0.172 × 0.172 mm^2^) and a microfocus X‐ray radiation source (λ = 1.5419 Å). For XRR measurement, the sample‐to‐detector distance was determined to be 1350 mm. The slits were adjusted to have a beam of 0.5 × 0.5 mm^2^ at the sample position. For this particular thickness analysis, MOF@PEDOT composite film is formed by spin coating as previously described on a polycrystalline silicon substrate.

#### SEM Analysis

SEM technique was used in order to characterize the morphology of the nanofilm composites. Images were acquired using a FEI Quanta 200 apparatus.

#### TGA

TGA (TA Instruments) was employed to access the relative composition of the native MOF (MIL‐101(Cr)) and the amino‐functionalized MOF (MIL‐101(Cr)‐NH_2_). Samples were measured with TGA using a heating rate of 10 °C min^−1^ from 25 to 800 °C under 50 mL min^−1^ N_2_ flow.

#### Z‐Potential Measurements

Surface Zeta potential measurements were performed at 25 °C for a fixed concentration suspension of MOF in a 1 × 10^3^ m KCl solution for increasing IMZ concentrations in a Malvern's Zetasizer Nano ZS.

#### Electrical Impedance Spectroscopy Measurements

The electrical impedance was measured with frequency sweeps using an Impedance analyzer (Reference 600, Gamry). The signal was sinusoidal of 20 mV_RMS_ amplitude and a frequency ranged from 0.1 Hz to 10 kHz. Each sample was cut in rectangles with dimensions of 20 mm × 5 mm and contacted on their opposite extremes using a bipolar configuration. The electrical contacts on the samples were fabricated using silver conducting ink (Electroquímica Delta). 40 µL of different solutions was added on top of the samples conductive path avoiding the contact with the silver paste (see Figure 2B). Petroleum jelly was manually applied on both sides of the silver contacts to confine the area of the liquids in contact with the composite surface. This strategy was previously reported in chlorine‐free sensors as suitable to avoid contact of solutions measured with silver electrical contacts.^85^

#### Pesticide Sensing

Imazalil (ACS, Supelco Analytical) stock solutions were prepared daily on 0.1 m KCl. After the addition of a 0.1 m KCl droplet, the electrical impedance scans were measured on the PEDOT and MOFs@PEDOT films repeatedly until the stabilization of the signal (approximately three to five scans). Then, the droplet was dried under N_2_ flow and the pesticide solution containing IMZ solved in 0.1 m KCl was added and the impedance measured until an equilibrated response was achieved (always reached in less than 30 min). For the IMZ calibration plot, the pesticide was added in increasing concentrations ranging from 0.015 to 1 ppm.

## Conflict of Interest

The authors declare no conflict of interest.

## Supporting information

Supporting Information
