# Supplementary material for: MOF@PEDOT Composite Films for Impedimetric Pesticide Sensors
Source: Glob Chall. 2020 Jan 8;4(2):1900076. doi: 10.1002/gch2.201900076 (PMC7001120; doi:10.1002/gch2.201900076)
Supplement: Supplementary file 1 — Supporting Information [file GCH2-4-1900076-s001.pdf]

Copyright WILEY-VCH Verlag GmbH & Co. KGaA, 69469 Weinheim, Germany, 2020.

Global Challenges
Open Access

Supporting Information

for *Global Challenges,* DOI: 10.1002/gch2.201900076

MOF@PEDOT Composite Films for Impedimetric Pesticide Sensors

Luciano D. Sappia, Jimena S. Tuninetti, Marcelo Ceolín, Wolfgang Knoll, Matías Rafti, and Omar Azzaroni*

Copyright WILEY-VCH Verlag GmbH & Co. KGaA, 69469 Weinheim, Germany, 2018.

Supporting Information

MOF@PEDOT Composite Films for Impedimetric Pesticide Sensors

Luciano D. Sappia, [‡] *Jimena S. Tuninetti,* [‡*] *Marcelo Ceolín, Wolfgang Knoll, Matías Rafti and Omar Azzaroni*

Dr. L. D. S., Dr. J. S. T., Dr. M. C, Dr. M. R. and Prof. O. A.

Instituto de Investigaciones Fisicoquímicas Teóricas y Aplicadas, Departamento de Química, Facultad de Ciencias Exactas, Universidad Nacional de La Plata, CONICET, CC 16 Suc. 4, La Plata, B1904DPI, Argentina.
E-mail: jtuninetti@inifta.unlp.edu.ar

Prof. O. A.

CEST-UNLP Partner Lab for Bioelectronics, Diagonal 64 y 113, La Plata (1900), Argentina.

Prof. W. K.
CEST - Competence Center for Electrochemical Surface Technologies – Konrad Lorenz Strasse 24, 3430 Tulln, Austria.

Austrian Institute of Technology - Donau-City-Strasse 1, 1220 Vienna, Austria.

[‡]These authors contributed equally to this work.

Keywords: conductive polymers; impedance spectroscopy; pesticide sensor; imazalil; Metal-organic frameworks; PEDOT

Section S1: Pesticide legislation and MRLs

In Table S1, some examples of MRLs are described according to EU Regulation (EC) N°396/2005 and its update regarding MRLs (N°750/2010), which is applicable from August 2010 up to date.

Table S1: Examples of MRL for imazalil in fresh or frozen food in the EU[14]

Fruits (fresh or frozen), tree nuts	Maximum residual limit (MRL) / ppm
Citrus fruits (grapefruits, oranges, lemons, limes, mandarins, others)	5
Potatoes	3
Bananas, melons, pome fruits (apples, pears, quinces, others)	2
Teas, coffee, herbal infusions, cocoa and carobs	0.1*
Berries and small fruits (grapes, strawberries, cane fruits, others)	0.05*
Stone fruits (cherries, peaches, plums, others)	0.05*
Tree nuts (coconuts, hazelnuts, pecans, pine nut kernels, pistachios, others)	0.05*
Meat-based food, milk, eggs, honey	0.05*

(*) Indicates lower limit of analytical determination in food by HPLC/GC-MS.

Table S2: Citrus* maximum residue limit (MRL) worldwide

**Country/region**	**ppm ($mg.kg^{-1}$)**
Australia	10
Codex**	5
Canada	5
China	5
Singapore	5
Japan	5
USA	10
Taiwan	5
Indonesia	5
South Korea	5
European union	5
Hong Kong	10

(*) Including oranges, grapefruit, mandarins, and lemons.

**The Codex Alimentarius, or "Food Code" is a collection of standards, guidelines, and codes of practice adopted by the Codex Alimentarius Commission. The Commission, also known as CAC, is the central part of the Joint FAO/WHO Food Standards Programme and was established by FAO and WHO to protect consumer health and promote fair practices in food trade. FAO= Food and Agriculture Organization of the United Nations. WHO= World Health Organization.

A recent study in Cyprus reported a multi-residue analysis of pesticide residues in fruits and vegetables (commercial food samples were recollected during 2016) using gas and liquid chromatography coupled with mass spectrometry (GC–MS/MS and LC-MS/MS) as the reference analytical method. The results showed that 62.4 % of the food samples analyzed were contaminated with pesticide residues, whereas 38.5 % of the samples contained more than one pesticide. In addition, 6.7 % of the samples exceeded the MRLs of the regulation EU 396/2005.[84]

Section S2: MOFs physicochemical characterization

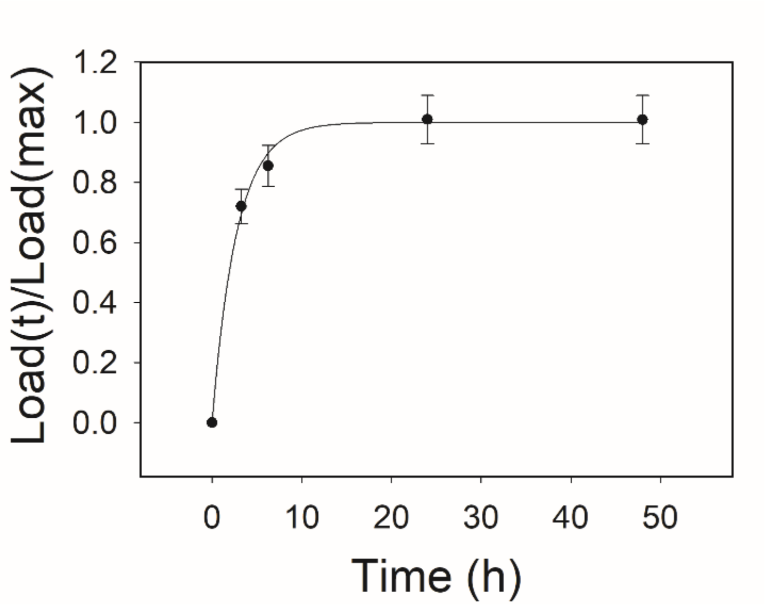

Figure S1. Kinetic of adsorption for IMZ on MIL-101(Cr)

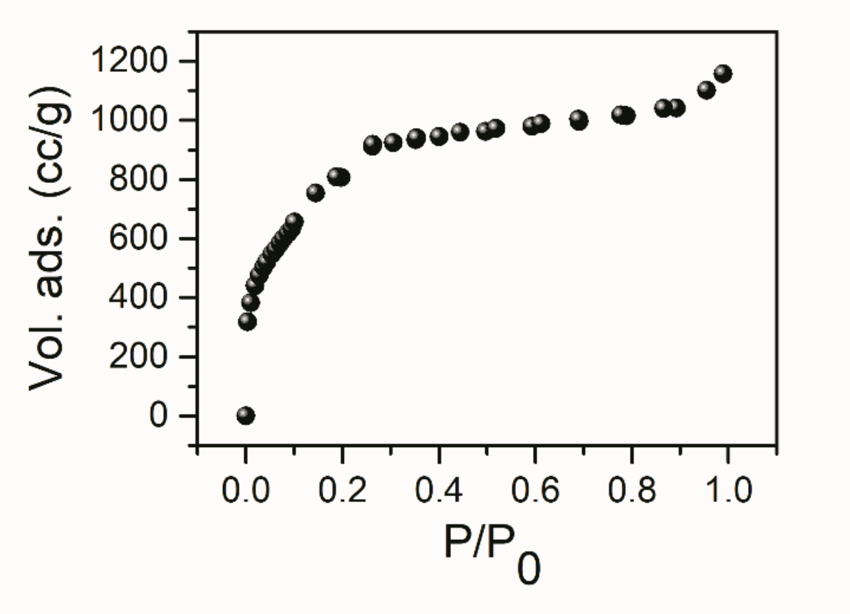

Figure S2. Adsorption N_2 Isotherm for MIL-101(Cr)

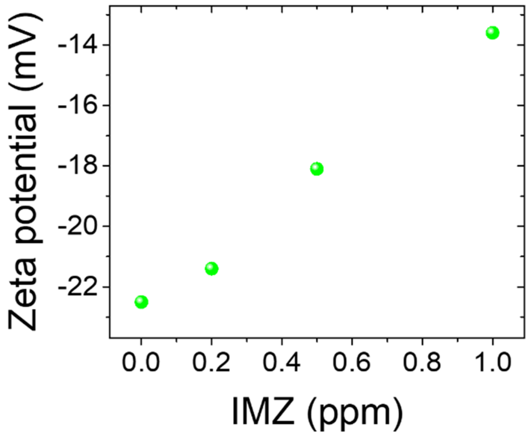

Figure S3. Zeta potential measurements for increasing IMZ concentrations added to an initial MOF-NH_2 suspension in 1 mM KCl.

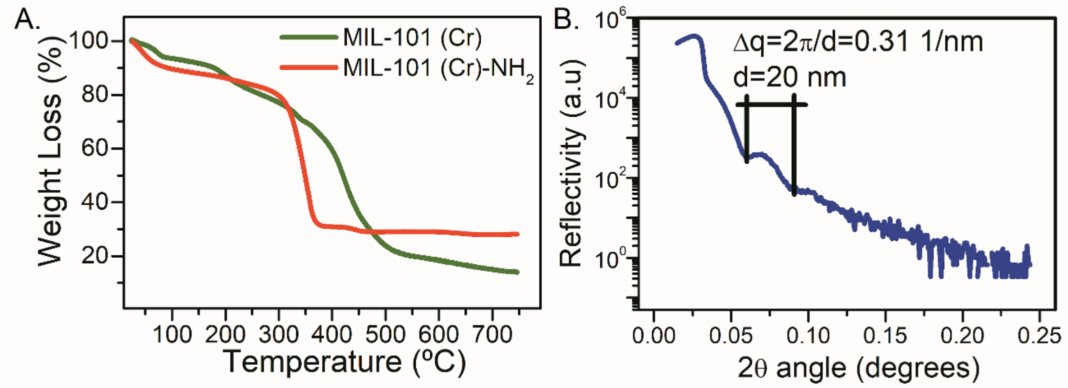

Figure S4. (A) Thermogravimetric analysis of MIL-101(Cr) and MIL-101(Cr)-NH_2. And (B) XRR thickness analysis for PEDOT film on the silicon surface.

The thickness of a smooth and transparent thin film can be analyzed by measuring the reflection of X-rays around the critical angle. When X-rays are applied below the total reflectance critical angle, they penetrate in the sample reflecting a fraction in each interphase with a characteristic electronic density. The interference between the reflected rays creates a diffraction pattern, which can be used to determine parameters of the layers, such as thickness, density and interfacial roughness without having into account the crystallinity of the film. Therefore, the thickness of the film can be determined from the experimental data by analyzing the distance between the first two minima of the reflectivity response in the nanometer range.

Section S3: Modeling of the impedance spectroscopy data and electrical equivalent circuits

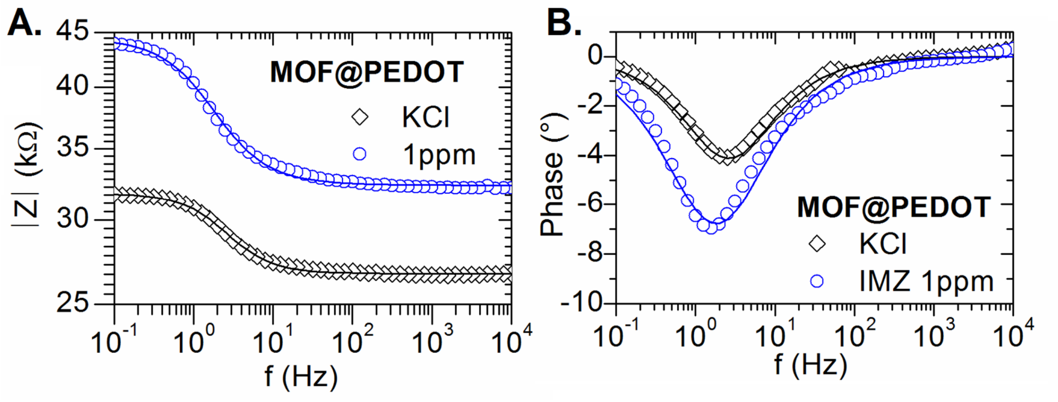

Figure S5. Experimental (circles) and fittings (continuous lines) of impedance module (A) and phase (B) response of the MOF@PEDOT film upon the addition of KCl 0.1 M and imazalil 1 ppm.

Fittings of the impedance spectroscopy measurements:

The inductive contribution of the wires at high frequencies was not taken into account in the electrical model, because it is only dominant at frequencies above 1 MHz.[85]

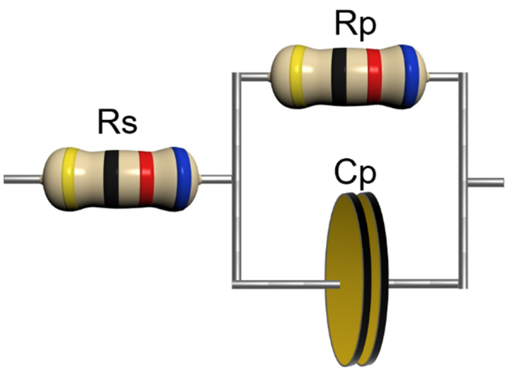

Scheme S1. The equivalent electrical circuit to model the impedance spectroscopy measurements was composed of a resistance Rs (modeling the electronic conduction path through the films) in series with an RC parallel circuit (modeling the charge transfer and accumulation between the polymer chains upon the addition of KCl solutions)

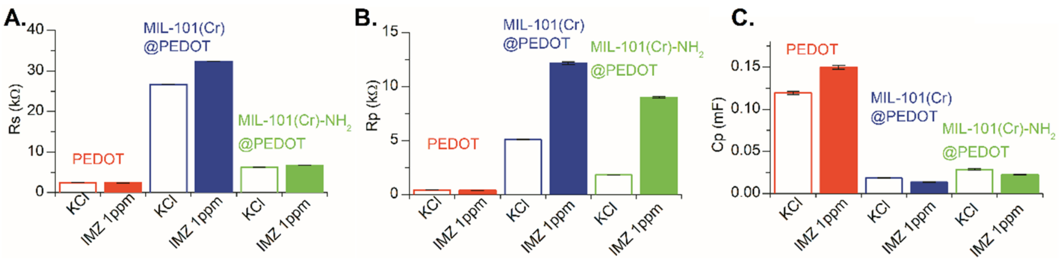

Figure S6. Representation of the electrical equivalent elements of the fittings upon the addition of 1 ppm KCl and IMZ. Rs (A), Rp (B), and Cp (C) are represented for PEDOT, MOF@PEDOT and MOF-NH_2@PEDOT films. The inset in (C), shows the equivalent circuit used to fit the experimental data.

Table S3. Percentage change in the passive elements from the equivalent electrical circuit used for modeling the impedance module of the pristine PEDOT, the MOF- and MOF-NH_2@PEDOT films after the addition of 1 ppm IMZ

Samples	%ΔRs	%ΔRp	%ΔCp
PEDOT	-5%	-12%	+25%
MOF@PEDOT	+21%	+139%	-27%

MOF-NH_2 @PEDOT	+7%	+394%	-22%

a) Percentage changes are calculated from the difference of the impedance module response between the addition of 1 ppm IMZ and 0.1 M KCl

Section S4: MOF@PEDOT interactions with IMZ and calibration plots

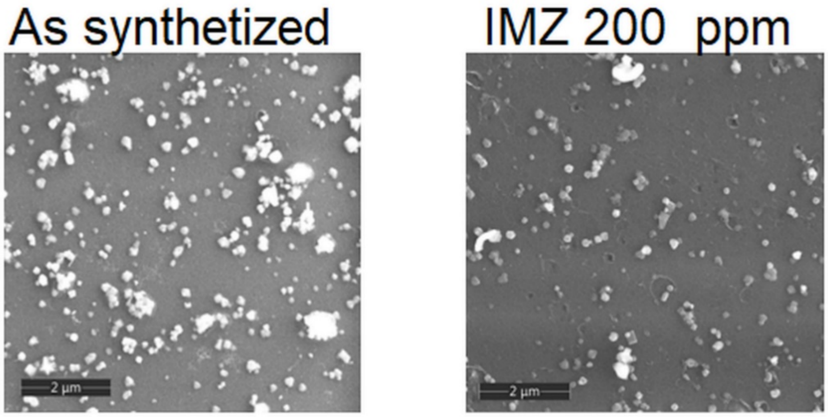

Figure S7. SEM of MIL-101(Cr)-NH_2@PEDOT before and after the incubation with IMZ 200 ppm in KCl 0.1M (Scale bar 2 µm)

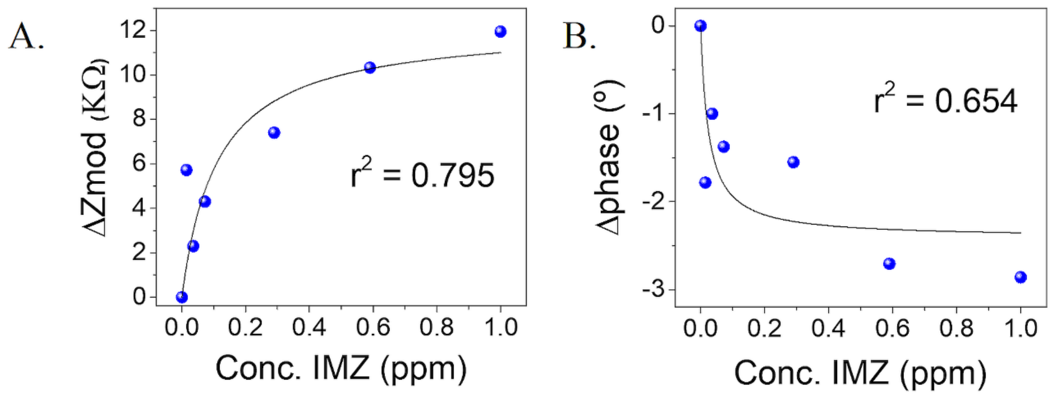

Figure S8. Imazalil response in MOF@PEDOT system in (A) Zmod and (B) phase

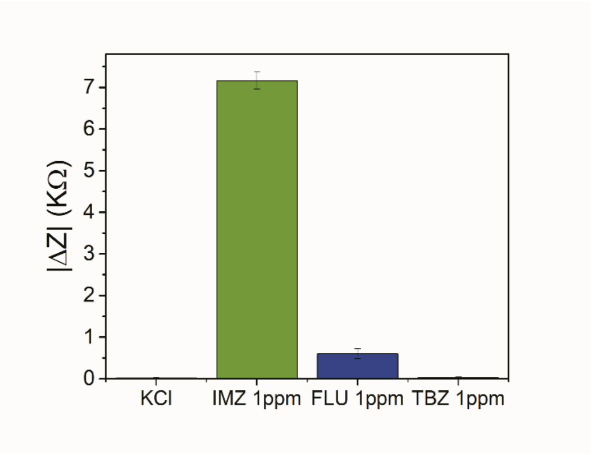

Figure S9. Specific response of the MOF-NH2@PEDOT composite due to Imazalil *vs.* Fluconazole (FLU) and Thiabendazole (TBZ) at 1 ppm evaluated as the modulus of the impedance at 0.2 Hz (n=3 different electrodes)

References

(1) European Commission. EU Pesticides Database - European Commission. Online. 2017.

(2) Constantinou, M.; Louca Christodoulou, D.; Constantinou, P.; Hadjigeorgiou, M.; Klavarioti, M.; Hadjiloizou, P.; Kika, K.; Kourouzidou, O. Multi-Residue Analysis of Pesticide Residues in Fruits and Vegetables Using Gas and Liquid Chromatography with Mass Spectrometric Detection. Accredit. Qual. Assur. 2018, 23 (3), 145–175. https://doi.org/10.1007/s00769-018-1320-1.

(3) Knipper M.a Parisi, J. . C. K. . W. C. . B. C. J. . D. V. .; Knipper, M.; Parisi, J.; Coakley, K.; Waldauf, C.; Brabec, C. J.; Dyakonov, V. Impedance Spectroscopy on Polymer-Fullerene Solar Cells. Zeitschrift fur Naturforsch. - Sect. A J. Phys. Sci. 2007, 62 (9), 490–494.
